# Evaluation of the In Vitro Permeation Parameters of Topical Diclofenac Sodium from Transdermal Pentravan^®^ Products and Hydrogel Celugel Through Human Skin

**DOI:** 10.3390/ph18060810

**Published:** 2025-05-28

**Authors:** Urszula Adamiak-Giera, Michał Gackowski, Joanna Szostak, Tomasz Osmałek, Damian Malinowski, Anna Nowak, Anna Machoy-Mokrzyńska, Maciej Miernik, Mirosław Halczak, Maciej Romanowski, Anna Czerkawska, Monika Białecka

**Affiliations:** 1 Department of Pharmacokinetics and Therapeutic Drug Monitoring, Pomeranian Medical University in Szczecin, 70-111 Szczecin, Poland; joannaszostak99@gmail.com (J.S.); damian.malinowski@pum.edu.pl (D.M.); ania.czerkawska@wp.pl (A.C.); monika-bialecka@post.pl (M.B.); 2 Chair and Department of Pharmaceutical Technology, Poznań University of Medical Sciences, 60-806 Poznań, Poland; mgackowski4@gmail.com (M.G.); tosmalek@ump.edu.pl (T.O.); 3 Department of Cosmetic and Pharmaceutical Chemistry, Pomeranian Medical University in Szczecin, 70-111 Szczecin, Poland; anna.nowak@pum.edu.pl; 4 Department of Experimental and Clinical Pharmacology, Pomeranian Medical University in Szczecin, 70-111 Szczecin, Poland; anna.machoy.mokrzynska@pum.edu.pl; 5 Department of General and Oncological Surgery, Pomeranian Medical University in Szczecin, 71-252 Szczecin, Poland; maciej.miernik@pum.edu.pl (M.M.); miroslaw.halczak@pum.edu.pl (M.H.); maciej.romanowski@pum.edu.pl (M.R.)

**Keywords:** penetration skin, vehicles, diclofenac sodium, human skin

## Abstract

**Background:** Diclofenac is a phenylacetic acid derivative classified as a non-selective COX inhibitor. Similar to other NSAIDs, it is characterized by anti-inflammatory, antipyretic, and analgesic effects. Long-term therapy with diclofenac might also lead to severe gastrointestinal, renal, or cardiovascular systems disorders. Aim of the study was to compare own formulation prepared from pharmaceutical raw materials with ready-to-use diclofenac product. **Methods**: In the in vitro permeation experiments, human skin was excised from the abdomen of living patients as a result of plastic surgery. The transdermal semi-solid formulations were compounded using Pentravan^®^, a ready-to-use transdermal base and hydrophilic gel base (Celugel). In vitro Penetration Studies, HPLC analysis, optical microscopy imaging, and a spreadability test were conducted. Rheological analysis provided insights into flow behavior, structure, and thixotropy. **Results**: Combination of Celugel with diclofenac sodium and the addition of substances acting as absorption enhancers, e.g., menthol, may provide an interesting alternative for enteral drugs, especially in patients with multimorbidity and polypharmacy. **Conclusions**: Topical diclofenac sodium with of addition of permeation enhancers like menthol might provide higher drug concentrations in the surrounding tissues and better analgesic and anti-inflammatory effects in compare to commercially available product and may provide optimum effectiveness with minimal risk of adverse effects, particularly in elderly and polymedicated patients.

## 1. Introduction

Pain is one of the most frequently reported symptoms, especially among elderly patients [1]. The definition of pain given by the International Association for the Study of Pain (IASP) suggests that it is an unpleasant sensory and emotional experience correlated with actual or potential tissue damage [2]. Considering the duration of pain, we can distinguish between acute and chronic pain. While considering the mechanism of pain formation, three types of pain are known: nociceptive, neuropathic, or nociplastic pain [3]. Accordingly, various pain treatment possibilities exist, making the correct diagnosis a significant factor while introducing proper and individualized pain therapy [4]. Nociceptive pain is known to be the most reported and diagnosed type of pain.

As one of the most prescribed analgesics, we can define the non-steroidal anti-inflammatory drugs (NSAIDs) [5], which are characterized by analgesic, anti-inflammatory, and antipyretic effects [6]. NSAIDs help patients suffering from nociceptive pain caused by various conditions, including osteoarthritis, rheumatoid arthritis, or even menstrual cramps [6]. The NSAIDs’ mechanism of action focused on their inhibitory properties on the two isoforms of cyclooxygenase enzyme (COX): cyclooxygenase-1 (COX-1) and cyclooxygenase-2 (COX-2). Under physiological conditions, COX-1 might be detected mainly in human organisms [6,7], whereas, COX-2’s expression is usually influenced by inflammatory factors [6]. Furthermore, the NSAIDs’ selectivity of the COX isoforms’ inhibition is one of the bases for their classification [6].

COX is responsible for the production of intermediate compounds such as prostaglandins (PG), prostacyclins (PGI), and thromboxane (TXA), which are significant for the proper functioning of the human body [8]. PG are involved, for example, in the development of the inflammatory process and contribute to swelling, pain, and increased vascular permeability [9], whereas PGI are responsible for blood flow and platelet behavior regulation [10]. Moreover, PG produced mainly by COX-1 positively impacts the digestive tract, kidneys, and platelet functioning [6]. Thus, most NSAIDs’ side effects correlate with COX-1 inhibition [6].

The risk of side effects of NSAIDs increases not only with the duration of the therapy [11], but also among patients diagnosed with other diseases requiring pharmacological treatment. NSAIDs’ most common side effects are correlated with the dysfunction of gastrointestinal and cardiovascular systems or kidneys [11]. During therapy with NSAIDs, patients might suffer, for example, from dyspepsia, nausea, stomach ulcer, acute or chronic renal failure, or hypertension [11].

Due to relatively frequent reports of NSAIDs’ severe adverse reactions, the interest in topical administration of the drugs has increased as a safer alternative to the oral route [12]. The transdermal route has many other advantages compared to the traditional oral route. Firstly, the drug has direct access to the target site of pain, thanks to the direct penetration through the skin’s outermost layer—the *stratum corneum*—and can reach the therapeutic concentration in surrounding tissues [5,12]. Lower possibilities of side effects and interactions with other medicines are also significant [12]. Moreover, bypassing the liver’s first-pass effect, the transdermal route leads to increased drug bioavailability [12]. Topical administration is usually more accepted, especially among young and old patients, positively impacting patients’ compliance [12]. There is a gap between pharmaceutical compounding and commercially available products, so there is a need for a search of new topical formulations enhancing the skin penetration of NSAIDs.

The effectiveness of the local therapy depends on several factors, such as the patient’s skin condition, form, and lipophilicity of the drug’s or cream’s basis [5]. Pentravan^®^ Celugel can be an excellent new transdermal carrier that can ensure the better skin penetration of NSAIDs, such as diclofenac sodium. Research has proven that the new cream basis like liposomal Pentravan^®^ or hydrogel Celugel may have a significant influence on the drug permeability [5,13]

Commonly topically administered analgesics and anesthetics are lidocaine or capsaicin and NSAIDs such as ketoprofen or diclofenac [5]. Topical NSAIDs are used as ointments, gels, creams, or patches [5].

Diclofenac is a phenylacetic acid derivative classified as a non-selective COX inhibitor [6,13]. Cognately to other NSAIDs, it is characterized by anti-inflammatory, antipyretic, and analgesic effects [6]. Long-term therapy with diclofenac might also lead to severe gastrointestinal, renal, or cardiovascular systems disorders [14]. Regardless, due to its effectiveness, diclofenac is still one of the most common NSAIDs. Topical diclofenac and ketoprofen therapy are gaining popularity, which is why, on the pharmaceutical market, we can find many ready-to-use products containing this substance. However, the research proves that creams or gels prepared from pharmaceutical raw material are characterized by higher permeability of NSAIDs and, consequently, better therapeutic effectiveness [5]. This study aimed to compare the skin penetration of diclofenac sodium (DCF) from the prepared formulations containing permeation enhancers with a reference product available on the market. Subsequently, an analysis of the physical and mechanical properties of the prepared formulations was performed.

## 2. Results

**Figure 1:**
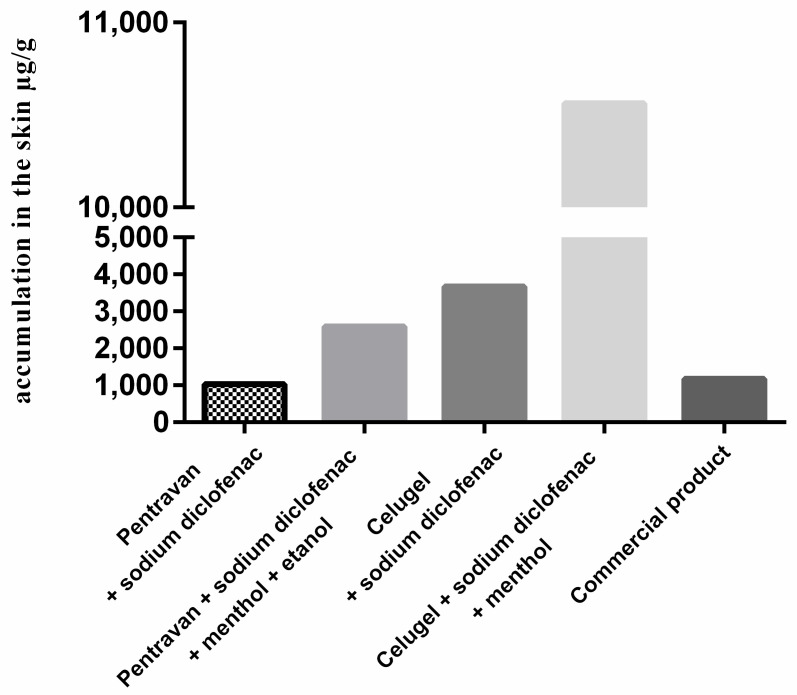
Accumulated DCF concentration in skin tissue [µg/g] after 24 h of the penetration study (mean ± SD, n = 3).

DCF permeates the human skin. The concentration of the test compound in the skin layer at the end of the experiment of the tested compound is presented in Figure 1.

The content in the acceptor fluid collected during 24 h permeation is summarized in Table 1.

DCF penetrated at most from F4 formulation. The cumulative amount of DCF permeated during the 24 h was 10,563.33 µg·cm^−2^. The results obtained with the Franz diffusion cells show that the highest concentration of DCF was recorded after 24 h in acceptor fluid after application formulation F4: 43.82 μg/mL. The second highest result of 3.35 μg/mL was observed in the same sample after 4 h. The third highest result was 3.18 μg/mL, recorded after 24 h in acceptor fluid after application formulation F1. However, the DCF was not detectable in this sample until 5 h after the beginning of the study. Comparing the sample results with formulations F3 and F2, we notice that after 24 h, the concentration of DCF in the first sample is lower (0.91 μg/mL) than in the second one (1.31 μg/mL). Nonetheless, the recorded concentrations of DCF after 1, 2, 3, 4, and 5 h were higher in the sample F3. The results of the commercial preparation sample were lower than those of all the other samples except sample F1.

The following results concern accumulation in human skin. The highest DCF concertation of 10,563.33 μg/g of skin was recorded in the sample F4. The second highest accumulation in the skin was determined in the sample with just formulation F3 and was almost three times lower than the first one (3671.33 μg/g of skin). The lowest skin accumulation (1033.13 μg/g of skin) was observed in the sample F1.

The release profiles of the active substance—graphs showing the dependence of the released amount of DCF on time—are provided in the Supplementary Materials (Figure S1).

F2 and F4 demonstrated superior permeation and were selected for further structural and rheological analysis. These included optical microscopy imaging, spreadability tests, and rheological measurements.

### 2.1. Optical Microscopy Imaging

**Figure 2:**
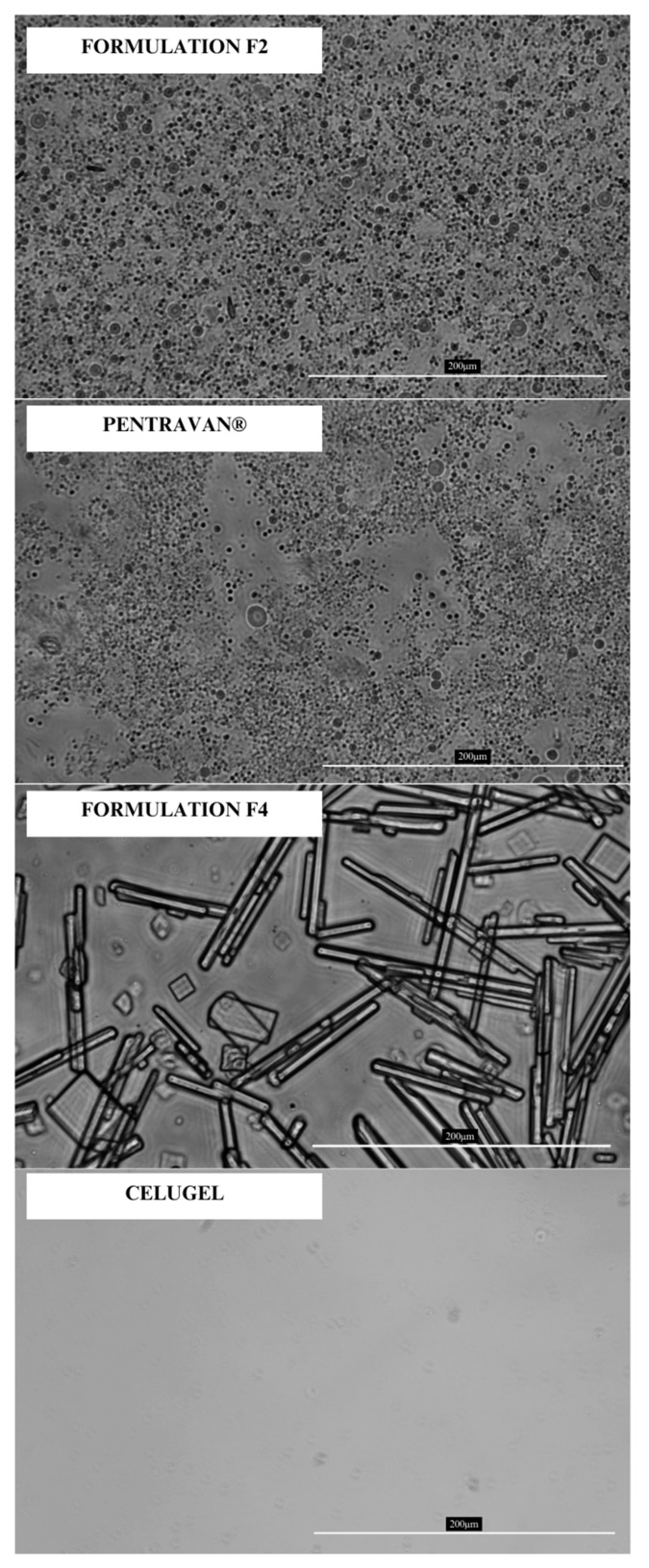
Optical microscopy images of formulations F2, F4, Pentravan^®^, and Celugel (magn. 40×).

Figure 2 presents images obtained during the optical microscopy analysis of Formulations F2, F4, Pentravan^®^, and Celugel.

### 2.2. Spreadability Test

The texture profiles obtained during the spreadability test are presented in Supplementary Materials (Figure S2).

Table 2 presents the values of the test parameters that were determined.

The study confirmed that the formulation F4 exhibited a more compact structure than F2. Both firmness and spreadability displayed higher values in the F4 formulation, while adhesiveness and adhesion force were lower than those observed for the F2 formulation.

### 2.3. Flow Curves and Thixotropy

**Figure 3:**
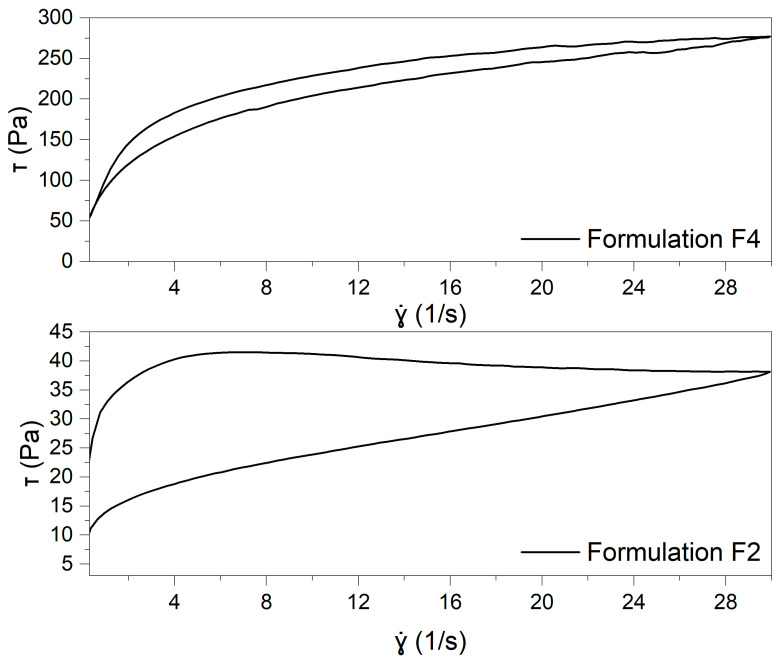
The flow curves of the formulations F2 and F4 with hysteresis loop.

The flow curves with the hysteresis loop of formulations F2 and F4 are displayed in Figure 3.

### 2.4. Stress Ramp Test

**Figure 4:**
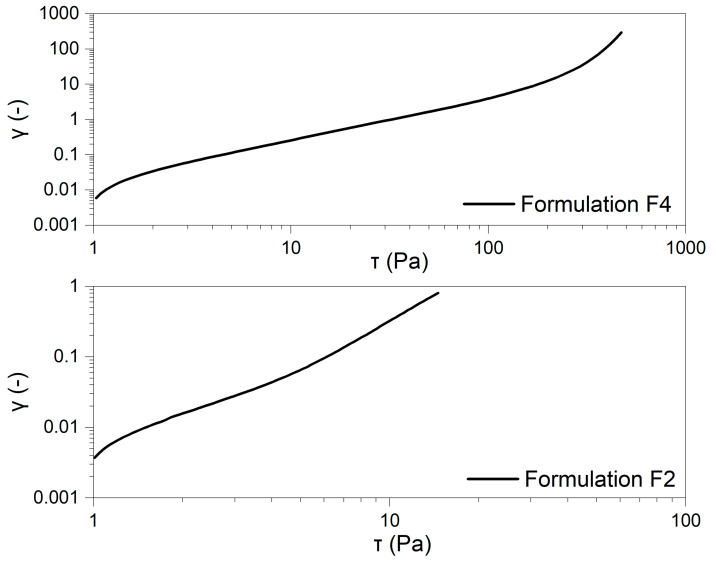
Double-logarithmic plots of sample deformation vs. shear.

The deformation vs. shear stress plots are presented in a logarithmic scale in Figure 4.

## 3. Discussion

NSAIDs are among the most commonly used groups of drugs in the treatment of nociceptive pain. Due to the rapidly growing number of patients requiring analgesic treatment, the search is ongoing not only for alternative routes of administration reducing the risk of adverse effects but also for innovative combinations of analgesics with different mechanisms of action containing absorption enhancers.

In the present study, we evaluated the in vitro human skin permeation of DCF from Pentravan^®^, a transdermal base, and Celugel, a hydrogel, without and with the addition of absorption enhancers. The addition of absorption promotors to selected formulations allows significantly higher drug concentrations to be achieved after topical application. Menthol is well known anesthetic and has beneficial analgesic effect, which is important in increasing the therapeutic range of preparations used.

In the first stage, we assessed the extent of DCF permeation from two formulations prepared using Pentravan^®^ and Celugel (F1, F2) without adding absorption enhancers compared to a commercial preparation. The cumulative mass of DCF after 24 h of the study in the case of the formulation F3 (3671.33 µg·cm^−2^) was higher as compared to F1 (1033.13 µg·cm^−2^) and the ready-to-use preparation (1174.03 µg·cm^−2^). These results demonstrate better release of DCF from a hydrogel to other bases, including Pentravan^®^, and the commercial preparation studied.

The results are consistent with findings from Folzer et al. and Manian et al. [15,16]. A study by Folzer et al. [15] demonstrated a better release of DCF from a commercial gel than from a solution. However, due to the limited availability of human skin, the authors investigated the permeation of DCF from different formulations using pig ear skin. The study showed that a topical diclofenac sodium gel is more suitable for achieving rapid pain control, whereas, DCF patches are more recommended for long-lasting pain relief. Manian et al. [16] found that the ointment formulation showed the lowest DCF release. The absence of the drug in the acceptor fluid indicates that the majority of the active substance remained on the skin surface, with only limited penetration into the epidermal and dermal layers. Among the tested formulations, the emulgel demonstrated superior skin retention. However, no statistically significant differences were observed between the gel and emulsion in terms of drug distribution within the skin layers. The enhanced skin permeation and retention seen with the emulgel may be attributed to the combined characteristics inherent to emulsion-based systems [16]. The study also demonstrated that the gel formulation studied exhibited the highest release among all the formulations analysed but showed comparably lesser skin retention and permeation than the emulsion formulation, in the case of which a lower drug release was observed. The study’s authors [16] noted that the gel or emulgel formulation may be more beneficial for pain management due to the ease of spreadability and elasticity and enhanced release and permeation. Similar to the present study, the study confirmed that depending on the intended therapeutic effect and site of action, the choice of a suitable base plays a vital role in the permeation and performance of semi-solid formulations.

Ready-to-use DCF preparations with a constant concentration and fixed composition are available in the pharmaceutical market. Our study showed that using a different base may significantly improve the absorption of topical diclofenac, and, thus, enhance the drug’s clinical effect. Similar conclusions were drawn concerning other analysed NSAIDs [17].

In the second stage of the study, we assessed the permeation of diclofenac sodium from Pentravan^®^ and Celugel with the addition of absorption enhancers, i.e., menthol and ethanol (F2, F4), as compared to Pentravan^®^ and Celugel without the addition of absorption enhancers (F1, F3). Adding menthol and ethanol to formulations led to significantly higher DCF penetration than formulations F1 and F3. The cumulative mass of DCF after 24 h of the study was (10,563.33 µg·cm^−2^) in the case of formulation F4 and (2590.00 µg·cm^−2^) in the case of F2. Both this study and the studies available in the literature demonstrate that gel bases show significantly better DCF release than emulsion bases [16]. This finding has very important implications for pain pharmacotherapy, as the effective release of active ingredients is an important factor affecting the bioavailability and penetration of drugs [16]. Our study showed that when menthol and ethanol were added to Pentravan^®^ and Celugel (F2, F4), DCF permeated through the skin to the acceptor fluid 30 min after the start of the study. The addition of different absorption enhancers to a base is known to be beneficial [18]. Our study demonstrated that adding menthol and ethanol to the bases studied significantly increased the release and permeation of DCF. We observed that diclofenac sodium was released from formulation F2 30 min after the start of the study. In contrast, for the formulation F1, DCF was only detected in the acceptor fluid 5 h after the start of the study.

Experimental temperature (37 °C) chosen for the study may have influenced the extent of API penetration through the skin, thereby affecting the final obtained values. It is important to emphasize that an increase in the temperature at which the permeation test is conducted enhances the penetration of the API [19].

Topical products have been developed to reduce the potential systemic effects that have been reported with orally administered drugs and to locally deliver the active drug to the site of injury for pain relief. Topically applied drugs present a promising alternative to oral medications, particularly in the management of knee osteoarthritis, where topical NSAIDs are recommended as a first-line option before oral therapy is considered. Their therapeutic effect depends on the drug’s ability to diffuse through the skin and reach deeper tissue layers. Several variables influence this process, including both the physicochemical properties of the active substance and the characteristics of the formulation. Research indicates that, following topical administration, the drug is capable of traversing the skin barrier and reaching underlying tissues, often resulting in higher concentrations in muscle compared to plasma levels observed with oral intake. Although the exact concentration of diclofenac required to effectively inhibit COX-2 remains somewhat uncertain, clinical evidence confirms its efficacy when applied topically. Patients commonly experience meaningful pain reduction in mild to moderate osteoarthritis, surpassing placebo effects and aligning with the outcomes of oral diclofenac therapy. Coupled with a more favorable safety profile, these findings support the use of topical diclofenac as a preferable alternative to systemic administration [20].

No studies in the literature compare the release of DCF from different bases containing additional absorption enhancers. Menthol is known to be used as an absorption enhancer. Moreover, it has analgesic effects, which may further enhance the pharmacological effect of the topical preparation used.

We also observed that the ready-to-use commercial preparation studied displayed a considerably lower transdermal flux of DCF compared to formulations F2, F3, and F4. The cumulative mass of diclofenac sodium after 24 h of the study in the case of the ready-to-use commercial preparation was 1174.03 µg·cm^−2^.

These results are consistent with previous research on the permeation of ketoprofen and ibuprofen and its derivatives from Pentravan^®^ [5,17]. As in the case of DCF, adding absorption enhancers significantly increased the permeation of the NSAIDs studied.

An analysis of the cumulative mass of DCF in the acceptor fluid collected at particular intervals showed that it took only 30 min for the drug to be released in significant amounts from the bases studied. In the case of NSAIDs, rapid skin permeation is beneficial, as it produces rapid therapeutic effects. The increased absorption within a shorter period results in a more rapid reduction in inflammation in the underlying tissues [21].

Evaluating in vitro penetration is a very important step in choosing a suitable dose and determining the composition of a vehicle for topical preparation. The level of active ingredient penetration may vary depending on the physicochemical properties of the compounds and the vehicle used [17,22].

In the last stage of the study, we conducted a spreadability and rheological study and took microscopic images. Microscopy imaging was performed to assess whether the liposomes present in the commercial Pentravan^®^ base remained intact during the preparation of the F2 formulation. This evaluation was critical because adding 5% ethanol (96% *v*/*v*) could potentially disrupt the liposomal structures [23]. Microscopic observations confirmed the presence of liposome-like structures in both the pure base and the final formulation. Additionally, optical microscopy enabled the examination of menthol crystallization in the F2 and F4 formulations. No crystalline structures were detected in the F2 formulation, whereas the F4 formulation exhibited numerous crystals of varying shapes and sizes. However, the majority of the observed structures were acicular and longitudinal. The evaluation of how menthol precipitates may potentially affect the permeation of the API through the skin has not yet been addressed in the literature, according to the authors’ knowledge. Due to their high water content and the addition of ethanol as a co-solubilizer, both bases enabled the complete dissolution of DCF, resulting in a solution-based system. Microscopic analysis revealed no presence of DCF crystals in any of the formulations.

Spreadability refers to a product’s ease of distribution, closely related to its firmness. A product that spreads more easily generally has lower firmness. Optimal spreadability is desirable for cosmetic and pharmaceutical formulations, such as ointments and creams. The device measures the force required to immerse the probe into the sample during the test. The work performed during this immersion serves as a quantitative measure of spreadability [24].

In addition to spreadability, the test determines firmness and adhesion-related parameters: adhesiveness and adhesive force [25]. These parameters help assess the formulation’s behavior during application and its potential ease of extrusion from a container, such as a tube. However, it is essential to note that the measurements were performed using an acrylic probe, meaning the results cannot be directly extrapolated to physiological conditions.

The study demonstrated that the F4 formulation exhibits a significantly more compact structure than the F2 formulation, a finding further supported by visual assessment. The greater firmness of the F4 formulation indicates that higher force is required to immerse the probe, which could translate into increased effort needed for product extrusion. Conversely, the lower firmness of the F2 formulation suggests a greater risk of unintentional leakage from the container. The F2 formulation also displayed superior spreadability, making it easier to apply than the F4 formulation. However, determining which formulation dependent on therapeutic objectives and formulation stability. While high spreadability and low firmness may enhance the application, they might also indicate insufficient retention on the skin. To complement the spreadability analysis involving normal forces, rheological studies examining the effects of shear forces can provide further insights.

Rheological studies provided insights into the tested formulations’ flow behavior, structural integrity, and thixotropic properties. The analysis allowed for assessing yield stress, viscosity profiles, and structural recovery, offering a comprehensive understanding of the formulations’ mechanical stability and potential application properties.

As illustrated in Figure 4, the flow curves demonstrate that both formulations exhibit pseudoplastic (shear-thinning) behavior, indicating a reduction in viscosity as the shear rate increases. The curves were fitted with the Windhab’s model (Equation (1)):

(1) $$\tau =\tau_{0}+\eta_{\infty }*\dot{\gamma }+\left(\tau_{1}-\tau_{0}\right)*(1-e^{-\frac{\dot{\gamma }}{{\dot{\gamma }}^{*}}})$$

where τ is the shear stress, τ_0_ is the shear stress at the zero point, η_∞_ is the infinite viscosity, $\dot{\gamma }$ is the shear rate, τ_1_ is the hypothetical yield stress, and ${\dot{\gamma }}^{*}$ represents the shear rate corresponding to the infinite viscosity [26]. The parameters of the model fitted to the formulation are presented in Table S1 (Supplementary Material).

Comparing the Windhab model parameters for formulations F2 and F4 reveals notable differences in their flow behaviors. The F4 formulation exhibits a more structured network, with higher yield stress and infinite viscosity, indicating greater resistance to deformation under shear. In contrast, F2 demonstrates weaker structural properties, reflected in lower yield stress and viscosity values. These findings suggest that F4 forms a more rigid structure, while F2 has more pronounced shear-thinning properties. A detailed description of the model parameters can be found in the Supplementary Materials.

Thixotropic properties were evaluated by calculating the thixotropy breakdown coefficient (Kd), defined as the ratio between the hysteresis loop area and the area under the ascending flow curve. This metric—also referred to as the relative hysteresis area—enables a more precise comparison between formulations with significantly different viscosities. The Kd value reflects the amount of energy required to disrupt the internal structure of the sample. It is worth noting, however, that at elevated shear rates, the accuracy of this measurement may be affected due to possible structural damage or sample loss caused by centrifugal forces [27]. The analysis of the thixotropic behavior of the tested formulations, based on the calculated Kd values, revealed significant differences between the samples. The F2 formulation exhibited a markedly higher Kd value (0.3193 ± 0.0017) than the F4 formulation (0.0857 ± 0.0101). This suggests that the internal structure of the F2 formulation undergoes greater breakdown under shear stress. In contrast, the F4 formulation demonstrated a lower Kd, indicating that its structure is more resistant to deformation and recovers more efficiently after removing shear stress. These differences may be attributed to the higher yield stress (τ₀) and overall viscosity (η∞) observed for the F4 sample, contributing to its greater structural stability.

**Figure 5:**
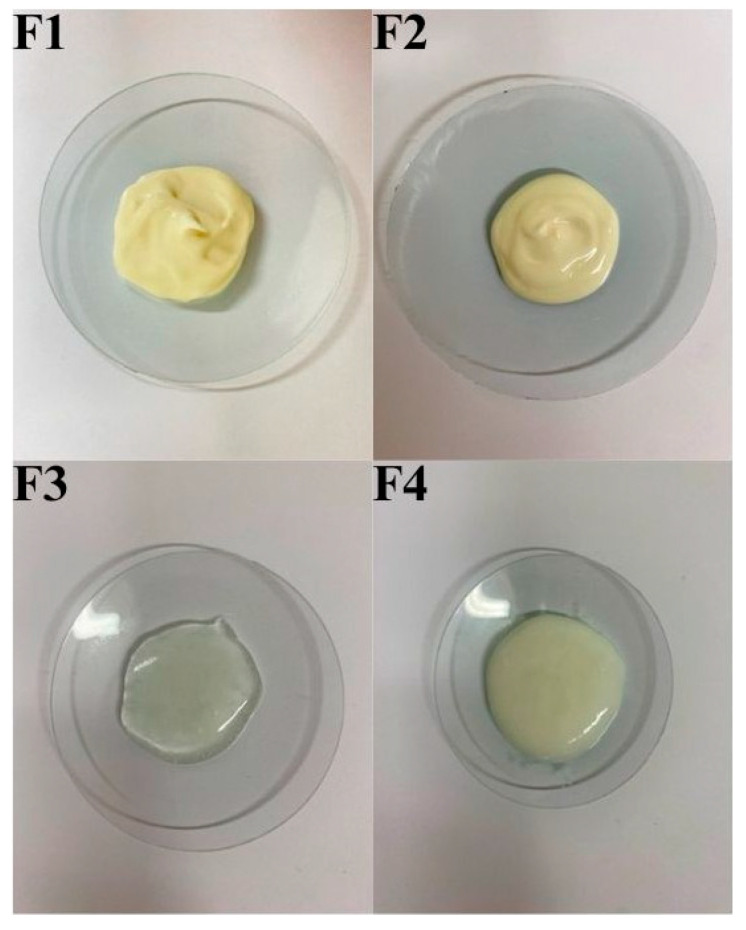
The comparison of formulation of the used F1, F2, F3, F4 vehicles.

The stress ramp test was carried out to assess the samples’ yield stress, representing the minimum force needed to initiate flow. Below this point, the material retains its elastic properties, while exceeding it causes structural breakdown and flow to begin [28]. Establishing yield stress as a single, fixed value is complex, as it is highly influenced by the testing method and experimental conditions. Therefore, the stress level at which the material starts to yield is often described as the apparent yield stress [29]. This parameter is critical in product formulation and processing, affecting storage, handling, and overall performance. It also significantly determines the product’s texture, ease of application, and the conditions required for manufacturing operations such as mixing, pumping, extrusion, or filling. Various techniques and measurement setups exist for evaluating yield stress, with the choice depending on the formulation’s specific characteristics and intended use. Figure 5 shows the deformation versus shear stress plots on a logarithmic scale.

The obtained yield stress (τ₀) values indicate a significant difference in the structural integrity of the two formulations. The yield stress of the F4 (250.967 ± 1.021 Pa) is considerably higher than that of the F2 formulation (4.864 ± 0.233 Pa), suggesting that Celugel forms a much stronger and more rigid gel network. This implies that the F4 formulation requires a significantly greater force to initiate flow, which may affect its spreadability and ease of application. In contrast, the much lower τ₀ value for F2 indicates a weaker internal structure, making it more fluid-like.

Cutaneous application of transdermal drugs offers may offer an effective way to prevent or minimize the possible adverse effects associated with the administration of oral drugs [30]. Additionally, the use of an appropriate vehicle can significantly improve the percutaneous absorption of active pharmaceutical ingredients. In this study, we investigated the transdermal delivery of DCF from two different bases: Pentravan^®^ and Celugel. The results were also compared to those obtained from a commercially available formulation containing the same concentration of the active compound. Our findings suggest that both Celugel and Pentravan^®^ serve as highly effective transdermal carriers, enabling the fast skin penetration of analgesic and anti-inflammatory agents like diclofenac sodium. Our findings indicate that the combination of DCF with Celugel and absorption enhancers (F4) would provide the highest concentrations of the drug following topical application.

To date, there have been limited studies evaluating the release of drugs from modern carriers such as Pentravan^®^. In our previous work, we investigated the effect of Pentravan^®^ on the transdermal permeation of ibuprofen and its derivatives, specifically ion-pair complexes of ibuprofen with new L-valine alkyl esters [ValOR] [IBU]. For all the ibuprofen derivatives tested, as well as for pure ibuprofen, Pentravan^®^ demonstrated significantly enhanced permeation compared to commercial formulations [17]. Pentravan^®^ has also been used as an active ingredient carrier in a study on hormonal therapy, in which the permeation of a vaginal hormonal drug from Pentravan^®^ was analysed [12]. There have been no studies to date evaluating the permeation of DCF from modern ready-to-use bases—Pentravan^®^ and the Celugel hydrogel.

The conducted spreadability and rheological studies provided valuable insights into the mechanical behavior of the tested formulations. The results demonstrated that the choice of the vehicle significantly influenced their structural properties and response to external forces. The F2 formulation exhibited a more fluid-like nature, indicating lower resistance to deformation. In contrast, the F4 formulation formed a much more rigid network, requiring greater force to initiate flow.

Our innovative research demonstrated that Celugel and Pentravan^®^ may be excellent transdermal vehicles ensuring rapid penetration of analgesic and anti-inflammatory drugs, such as DCF. The fact that there are no multi-drug formulations with good pharmacokinetic parameters in the pharmaceutical market has prompted attempts to describe the most beneficial multidrug combinations with a synergistic effect. As a rule, pain, particularly chronic pain, is treated with a multimodal approach in which different MOAs of analgesic drugs are employed. It would seem that topical formulations in treating pain, such as bone and joint diseases, could follow the same principles. The present study showed that combining Celugel with diclofenac sodium and adding substances acting as absorption enhancers, e.g., menthol, may provide an interesting alternative for enteral drugs, especially in patients with multimorbidity and polypharmacy.

## 4. Materials and Methods

### 4.1. Materials

Pentravan^®^, DCF, menthol, ethanol were purchased from Fagron (St, Paul., MN, USA); hydrogel Celugel was from Actifarm (Warszawa, Poland); potassium dihydrogen phosphate, acetonitrile, methanol, and phosphate-buffered saline (PBS, Boston, MA, USA; pH 7.40 ± 0.05) were from Sigma-Aldrich (St. Louis, MN, USA). All reagents were of analytical grade. Commercially product (CP) contained diclofenac sodium and the additional substances: isopropyl alcohol, propylene glycol, liquid paraffin, copolymer of ammonium acryloyl dimethyltaurate and *N*-vinylpyrrolidone cetostearyl macrogol ether, sorbitan trioleate, water, and fragrance composi-tions.

### 4.2. Skin

In the in vitro permeation experiments, human skin was excised from the abdomen of living patients as a result of plastic surgery. The study was approved by the Ethical Committee of Pomeranian Medical University in Szczecin (KB0012/02/18). The skin for the experiment was prepared as in the previous studies [5]. The surgeons took small pieces of skin and cut appropriately using a dermatome to maintain the same conditions. The skin was always taken from the same parts of the body in order to preserve the thickness of the skin. Human error was eliminated because the material was always taken by the same operator, in the same department. After collection, the skin was properly protected and directly used for permeation tests. Each formulation was tested on samples from the same donor, with four donors.

### 4.3. Vehicles

The research involved four distinct topical preparations, each featuring a unique formulation. These transdermal semi-solid agents were prepared using two different bases: Pentravan^®^, a preformulated transdermal carrier, and Celugel, a hydrophilic gel base. Pentravan^®^ is classified as an oil-in-water (o/w) emulsion base, with water being its primary ingredient, constituting approximately 62% of the formulation. Despite its high water content, it has the appearance of a thick, yellowish cream and maintains a pH range between 4.0 and 5.5. The formulation of Pentravan^®^ includes a range of components such as the LIPOIL complex, butylhydroxytoluene, simethicone, urea, potassium sorbate, polyoxyethylene stearate, cetyl alcohol, stearyl alcohol, stearic acid, glycerol monostearate, benzoic acid, carbomer, and hydrochloric acid. In contrast, Celugel is a hydrophilic gel composed of water, glycerol, and hydroxyethylcellulose, which serves as the gelling agent.

DCF was added to the vehicles and various pharmaceutical raw materials, i.e., menthol and ethanol. DCF was placed in a mortar and mixed with a small amount of the vehicle. Next, the remaining vehicle parts were added in portions while gently mixing. Menthol was micronized with ethanol in a mortar; the vehicle was added in portions. Individual compositions were prepared according to pharmaceutical practice. For comparative purposes, commercial products labeled as CP were also analyzed. The composition of individual agents is presented in Table 3.

Figure 5 shows the appearance of the various preparations prepared in our study using Pentravan^®^ and Celugel substrates.

### 4.4. In Vitro Penetration Studies Through Human Skin

In vitro permeation studies were performed using Franz diffusion cells (Phoenix DB-6, ABL&E-JASCO, Wien, Austria) with diffusion areas of 1 cm^2^ and biological membrane (human skin). The volume of the acceptor chamber was 8 cm^3^, and it was filled with PBS solution (pH 7.4). In each diffusion unit, a constant temperature of 37.0 ± 0.5 °C was maintained, according to Milanowski et al. [31]. The content of the acceptor chamber was continuously stirred using a magnetic stirrer set to a constant speed of 200 RPM across all diffusion cells. Skin samples were mounted onto the donor chambers, with care taken to select only intact pieces of uniform thickness for the experiment. The permeation test was conducted over a 24-h period. Each donor chamber received 1 g of the test formulation. Samples from the acceptor fluid were collected at multiple time points: 0.5, 1, 2, 3, 4, 5, and 24 h after the start of the experiment. Following each collection, 0.4 mL of the acceptor fluid was withdrawn and replaced with an equal volume of buffer solution at the same pH. All experiments were carried out in triplicate (n = 3). Diclofenac sodium concentrations in the acceptor phase were quantified using the HPLC-UV method. The cumulative amount of diclofenac sodium permeated per unit area (µg·cm^−2^) was calculated from the measured concentrations. In vitro skin accumulation was measured analogously as described by Haq and Michniak-Kohl [32].

### 4.5. HPLC Analysis

DCF was performed by HPLC-UV (Agilent, Hongkong, China, HP 1100) using Hypersil Gold (C18) 100 mm × 3 mm column (Thermo Scientific™, Waltham, MA, USA). Mobile phase composed of 0.02 M potassium dihydrogen phosphate—acetonitrile—methanol (16/16/20, *v*/*v*/*v*) at 0.5 mL/min. The column temperature was set at 22 °C. The UV detection at 280 nm was employed, and the injection volume was 20 µL.

### 4.6. Statistical Analysis

Data are presented as mean ± standard deviation (SD), DCF cumulative permeation mass (µg·cm^−2^). The Shapiro–Wilk normality test was used to examine the distribution of data. Student’s *t*-test determined the statistical significance of differences. Statistical analysis was performed using STATISTICA PL, ver. 13.3 [StatSoft, Inc. Tulsa, OK, USA, 2016, STATISTICA-data analysis software system] software. Statistically significant differences (*p*-value) were assumed at *p* < 0.05.

### 4.7. Optical Microscopy Imaging

The microscopic images of the formulations F2, F4, Pentravan^®^, and Celugel were performed with Leica DM IL-Led (Leica, Wetzlar, Germany) with N Plan Fluor 40×/0.60 lens optical microscope equipped with JENOPTIK GRYPHAX ProgRes camera (JENOPTIK, Jena, Germany). The images were captured under a magnification of 40×.

### 4.8. Spreadability Test

The spreadability test was conducted using a texture analyzer equipped with cone-shaped attachments—Shimadzu AGS-X, 10N-10kN (SHIMADZU, Kyoto, Japan)—and operated with TRAPEZIUM X 1.52 software. The analysis was performed at ambient temperature, with each measurement repeated three times (n = 3) to ensure accuracy. The sample was placed in the female cone, and then the male cone was moved downward at a speed of 60 mm·min^−1^, immersed in the sample, and pressed against the female cone. The instrument measured the force required to immerse the probe into the sample. Afterward, the male probe was lifted upward, and the test was stopped when the sample completely detached from the probe.

### 4.9. Rheological Measurements

Rheological analysis was conducted using a HAAKETM RheoStress1 rotational rheometer (Thermo Electron Corp., Waltham, MA, USA). Temperature regulation was maintained via a HAAKETM DC30 thermostat equipped with a recirculating water bath. Measurements were performed using a titanium plate-plate setup (Φ 35 mm). Following a 10-min incubation at 25 °C, the samples were carefully applied to the lower plate using a spatula. The measuring gap was adjusted to 1.0 mm. Once the upper plate was lowered, any excess sample was gently removed, ensuring minimal unintended shear. A waiting time of 120 s was set between loading and measurement to allow for sample equilibration. The test temperature was maintained at 25.0 ± 0.2 °C throughout the procedure. Data collection and analysis were conducted using HAAKETM RheoWin^TM^ Job Manager and Data Manager 4.91.0021 software (Thermo Electron Corp., Waltham, MA, USA). Each measurement was performed on a fresh sample, with at least three replicates per formulation, and the average values of the parameters were recorded.

#### 4.9.1. Steady Shear Experiments

Flow curves and thixotropy:

Flow curves were generated by plotting shear stress (τ) as a function of shear rate (γ). The shear rate was varied from 0 to 30.0 s^−1^ in an increasing ramp, followed by a decreasing ramp. Each ramp was applied over 100 s, with no interval between them. Thixotropy was expressed as the ratio of the hysteresis loop area to the total area under the ascending curve—as relative hysteresis area (Kd).

#### 4.9.2. Stress Ramp Test (CS—Controlled Stress)

Shear stress-dependent measurements were conducted over different stress ranges: 1.0–500.0 Pa for the F4 formulation and 1.0–15.0 Pa for the F2 formulation. The assay duration was 60.0 s for F4 and 20.0 s for F2. The results were presented as strain versus shear stress on a logarithmic scale. Due to the significantly lower adhesiveness of the F2 sample, different measurement conditions were applied for each formulation to ensure reliable data acquisition.

## 5. Conclusions

The practical value of this study is that it demonstrates that a compounded topical diclofenac sodium-containing preparation might provide higher drug concentrations in the surrounding tissues and better analgesic and anti-inflammatory effects. The combination of diclofenac sodium with modern formulation substrates such as Pentravan^®^ and Celugel with the addition of absorption promoters would allow for significantly higher drug concentrations and better analgesic and anti-inflammatory effects of the drug after topical application than with a ready-to-use commercial formulation. Compounding allows using a topical drug with an optimum composition and concentration, containing a suitable base and absorption enhancers. Personalized treatment of pain using topical drugs may provide optimum effectiveness with minimal risk of adverse effects. The above studies should be performed in an in vivo model or in clinical trials to confirm the presented findings.

## Figures and Tables

**Table 1:** Diclofenac sodium permeation in the human skin (cumulated mass for DCF after application on the skin Pentravan^®^ and Celugel).

	Mean Concentration ± SD [µg/mL]	Diclofenac Sodium Cumulative Permeation Mass (µg·cm^−2^)	*p*-Value *
F1	104.07 ± 69.39	1033.13	0.8043
F2	259.00 ± 56.11	2590.00	0.0353
F3	485.16 ± 80.57	3671.33	0.0028
F4	1056.95 ± 114.49	10,563.33	0.0002
CP ^#^	117.57 ± 54.66	1174.03	reference

* student-*t* test; ^#^ CP—commercially product.

**Table 2:** The parameter values determined during the spreadability test are presented as the mean of three measurements (n = 3) ± SD.

Formulation	Adhesiveness [N × s]	Firmness [N]	Spreadability [mJ]	Adhesion Force [N]
F2	1.726 ± 0.262	0.125 ± 0.019	0.292 ± 0.048	0.064 ± 0.016
F4	5.427 ± 0.629	0.376 ± 0.067	0.612 ± 0.12	0.154 ± 0.025

**Table 3:** The composition of individual agents.

Ingredient	Pentravan^®^ and Diclofenac Sodium (F1)	Pentravan^®^, Diclofenac Sodium, Menthol and Ethanol (F2)	Celugel and Diclofenac Sodium (F3)	Celugel, Diclofenac Sodium, Menthol and Ethanol (F4)
Pentravan^®^	98.0	83.0	-	-
Celugel	-	-	98.0	83.0
Diclofenac sodium	2.0	2.0	2.0	2.0
Menthol	-	10.0	-	10.0
Ethanol 96% (*v*/*v*)	-	5.0	-	5.0

The amounts of components are expressed in g. Ethanol and menthol—permeation enhancers (Pharmacopeia concentrations); menthol is anesthetic and has beneficial analgesic effect, which is important in increasing the therapeutic range of preparations used.

## Data Availability

All data provided within this manuscript or in Supplementary Materials. The data presented in this study are available on request from the corresponding author.

## Supplementary Materials

The following supporting information can be downloaded at: https://www.mdpi.com/article/10.3390/ph18060810/s1, Figure S1. Time course of the permeation mass µg·cm^−2^ (X axis) of the diclofenac sodium in the skin during the 24 h (Y asis) penetration from F2 and F4. Figure S2. Texture profiles of the formulations plotted as a force against time. Table S1. Windhab model values for the tested formulations.

## Abbreviations

The following abbreviations are used in this manuscript:

NSAIDs	non-steroidal anti-inflammatory drugs
COX	cyclooxygenase enzyme
TXA	thromboxane
PG	prostaglandins
PGI	prostacyclins
DCF	diclofenac sodium
PBS	phosphate buffered saline
SD	standard deviation
CS	Controlled Stress
kD	relative hysteresis area
r	correlation coefficients
η	viscosity
τ	yield stress
