# Supplementary material for: Evaluation of the In Vitro Permeation Parameters of Topical Diclofenac Sodium from Transdermal Pentravan® Products and Hydrogel Celugel Through Human Skin
Source: Pharmaceuticals (Basel). 2025 May 28;18(6):810. doi: 10.3390/ph18060810 (PMC12195876; doi:10.3390/ph18060810)
Supplement: Supplementary file 1 [file pharmaceuticals-18-00810-s001.zip › pharmaceuticals-3598038-supplementary.pdf]

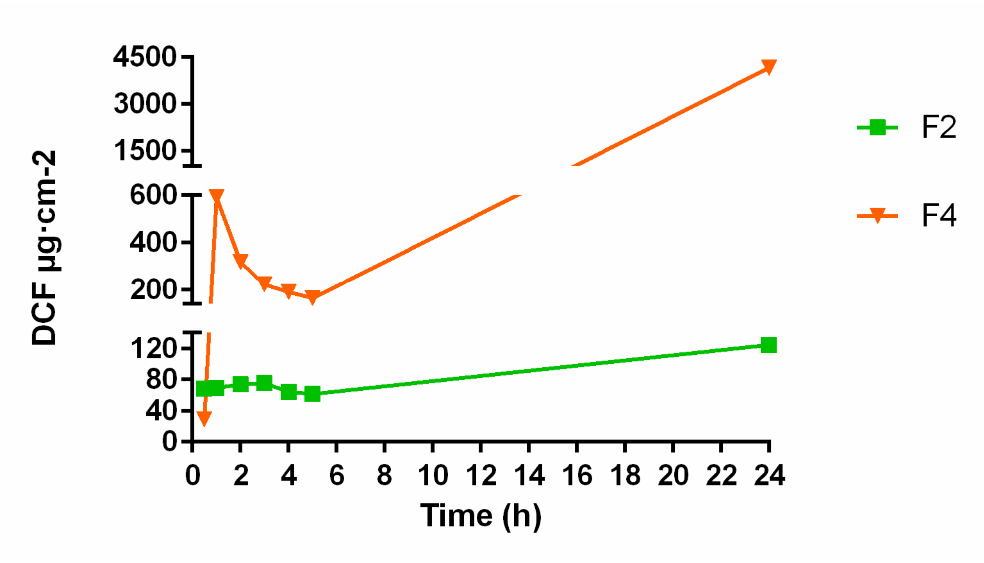

Figure S1. Time course of the permeation mass µg·cm^{-2} (X axis) of the diclofenac sodium in the skin during the 24 h (Y asis) penetration from F2 and F4.

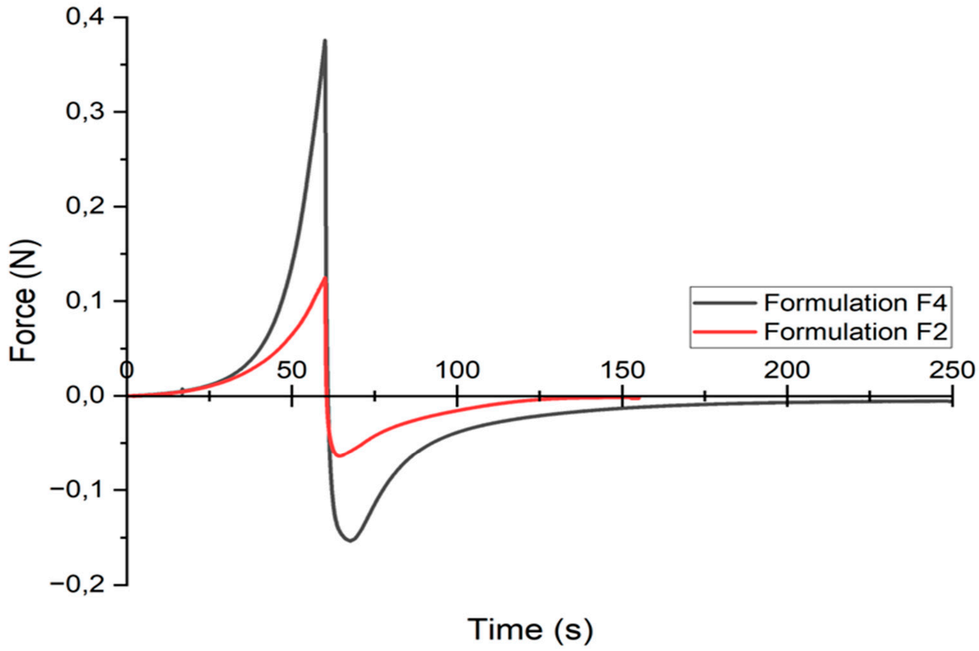

Figure S2 - Texture profiles of the formulations plotted as a force against time.

Table S1 - Windhab model values for the tested formulations.

Parameter	Formulation F2	Formulation F4
**Correlation coefficient (r)**	0.986±0.003	0.997±0.001
**τ_0**	21.72±1.782	46.787±2.271
**η_∞**	-0.161±0.016	2.369±0.096
**τ_1**	42.417±0.185	212.267±3.188
**$\dot{\gamma}^*$**	1.306±0.031	2.751±0.13
**Relative hysteresis area (Kd)**	0.3193±0.0017	0.0857±0.0101

The correlation coefficients (r) indicate a strong fit of the Windhab model to the experimental data, with values exceeding 0.98 for both formulations, confirming the model's suitability in describing their flow behavior. The τ_0 parameter, representing the shear stress at the zero point, was significantly higher for the F4 formulation (46.787±2.271 Pa) compared to the F2 formulation (21.72±1.782 Pa), suggesting a more structured network within the Celugel matrix. The infinite viscosity (η_∞) exhibited distinct differences between the two formulations. The obtained η_∞ value for formulation F2 is negative (-0.161±0.016 Pa • s), indicating potential measurement artifacts due to the extremely low viscosity of the sample. It is likely that the sample flowed out from between the plates at high shear rates, as evidenced by a slight bump in the initial ascending curve (Figure 4). Therefore, this result should be interpreted with caution. However, both rheological and spreadability analyses consistently confirm the weak structure of the formulation, supporting its pronounced shear-thinning properties. Despite measurement limitations, the experiment was conducted under identical conditions for both samples, allowing for a reliable comparison. In contrast, the F4 formulation maintained η_∞ value (2.369±0.096 Pa • s), implying greater resistance to deformation under increasing shear rates. The hypothetical yield stress (τ_1) was substantially higher for F4 (212.267±3.188 Pa) compared F2 (42.417±0.185 Pa), reinforcing the conclusion that F4 forms a more rigid structure requiring greater force to initiate flow. Finally, the shear rate ($\dot{\gamma}^*$) corresponding to the infinite viscosity, was also higher for F4 (2.751±0.13 s-1) than for F2 (1.306±0.031 s-1), further supporting the notion of a more cohesive internal structure in the Celugel-based formulation
